# Gray Matter Axonal Connectivity Maps

**DOI:** 10.3389/fpsyt.2015.00035

**Published:** 2015-03-06

**Authors:** Leonardo Bonilha, Ezequiel Gleichgerrcht, Travis Nesland, Chris Rorden, Julius Fridriksson

**Affiliations:** ^1^Department of Neurology, Medical University of South Carolina, Charleston, SC, USA; ^2^Department of Psychology, University of South Carolina, Columbia, SC, USA; ^3^Department of Communication Sciences and Disorders, University of South Carolina, Columbia, SC, USA

**Keywords:** connectome, magnetic resonance imaging, diffusion tensor imaging, structural networks

## Abstract

Structural brain connectivity is generally assessed through methods that rely on pre-defined regions of interest (e.g., Brodmann’s areas), thus preventing analyses that are largely free from *a priori* anatomical assumptions. Here, we introduce a novel and practical technique to evaluate a voxel-based measure of axonal projections connecting gray matter tissue [gray matter axonal connectivity map (GMAC)]. GMACs are compatible with voxel-based statistical approaches, and can be used to assess whole brain, scale-free, gray matter connectivity. In this study, we demonstrate how whole-brain GMACs can be generated from conventional structural connectome methodology, describing each step in detail, as well as providing tools to allow for the calculation of GMAC. To illustrate the utility of GMAC, we demonstrate the relationship between age and gray matter connectivity, using voxel-based analyses of GMAC. We discuss the potential role of GMAC in further analyses of cortical connectivity in healthy and clinical populations.

## Introduction

Magnetic resonance (MR) diffusion tensor imaging (DTI) can be used to reconstruct white matter water molecule diffusion pathways, which are considered to be the biophysical representations of axonal bundles and their myelin sheath (1, 2). The combination of data from DTI tractography and data from segmentation of gray matter tissue (derived from T1-weighted MR images) into anatomical regions of interest (ROIs) enables the quantification of white matter pathways connecting gray matter ROIs (3). The structural brain connectome, which is an individualized whole-brain map of white matter connectivity (3, 4), can thus be obtained by assessing the DTI connectivity between all possible combinations of gray matter ROIs.

Examining the brain connectome and its relationship with behavioral phenomena or neurological symptoms has become a popular way to address the association between brain structure and function (5–7). The brain connectome can be used to evaluate the effects of regional and global network organization as they relate to developmental and pathological processes associated with changes in connectivity (7).

One important limitation of the current methodology employed in connectome studies, however, is its strong dependence on anatomically pre-defined gray matter ROI parcellation atlases. In general, gray matter segmentation is performed based on the probabilistic subject’s gray matter map (composed of cortical and subcortical regions) fitted onto an anatomically defined atlas (8, 9). While there is an abundance of examples of gray matter atlases, it is well recognized that the division of the cortex into ROIs is a semi-arbitrary process, which typically does not directly represent functional or histological boundaries. Individual variability in cortical anatomo-functional localization, notably as it relates to more superficial and more variable sulci and gyri, may lead to cortical subdivisions that do not exactly represent equivalent functional areas across individuals.

Thus far, existing approaches designed to overcome this problem, such as cytoarchitectonic atlases (10–12)^1^, or custom-made functionally defined ROIs (e.g., from functional MRI data) can provide a limited coverage of the brain and are unable to provide a scale-free representation encompassing the whole brain.

In this study, we describe a new methodology aimed at overcoming this limitation. We demonstrate how whole-brain gray matter axonal connectivity maps (GMAC) can be generated from conventional connectome methodology, yielding a voxel-based representation of gray matter connectivity that are largely independent from parcellation atlases and compatible with voxel-based statistical analyses.

## Methods

### Subjects

We assessed 18 healthy individuals (mean age 40.5 ± 5.3 years, 8 males) recruited from the local community with no significant past medical history of neurological or psychiatric problems. This study was approved by the Institutional Review Board of the Medical University of South Carolina. All subjects signed an informed consent to participate in this study.

### MRI acquisition

Images were acquired on a Siemens 3 T Verio MRI scanner equipped with a 12-channel head coil. Two sequences were employed: (1) high-resolution T1-weighted image, with an isotropic voxel size of 1 mm (TR = 2250 ms, TE = 41 ms, FOV = 256 mm × 256 mm); and (2) diffusion-weighted images using two diffusion weightings (*b* = 0 and 1000 s/mm^2^) along 30 diffusion-encoding directions (TR = 10,600 ms, TE = 100 ms, FOV = 224 mm × 224 mm, parallel imaging factor of 2, slice thickness = 2 mm, and 60 axial slices, isotropic voxel size of 3 mm).

### Image processing

#### Overview

The initial preprocessing steps were similar to conventional steps employed in connectome reconstruction (5). We employed a probabilistic approach for DTI tractography (13). We chose to employ probabilistic tractography in this study, since it is theoretically capable of accommodating intra-voxel fiber crossings and complex fiber geometry (13, 14). Cortical seed regions for tractography were obtained from an automatic segmentation process employing FreeSurfer (15)^2^ [the Lausanne anatomical atlas, distributed as part of the Connectome Mapping Toolkit (9)]^3^. This initial atlas-based ROI segmentation is performed solely to provide starting points for tractography. The ROIs were transformed into each subject’s native DTI space using an affine transformation obtained with FMRIB Software Library (FSL)’s FLIRT (16). Probabilistic tractography was then performed using each one of the gray matter ROIs in diffusion space as a seed region, with the no-diffusion dMRI sequence (B0 image) as the inclusion mask for fiber tracking.

**Figure 1:**
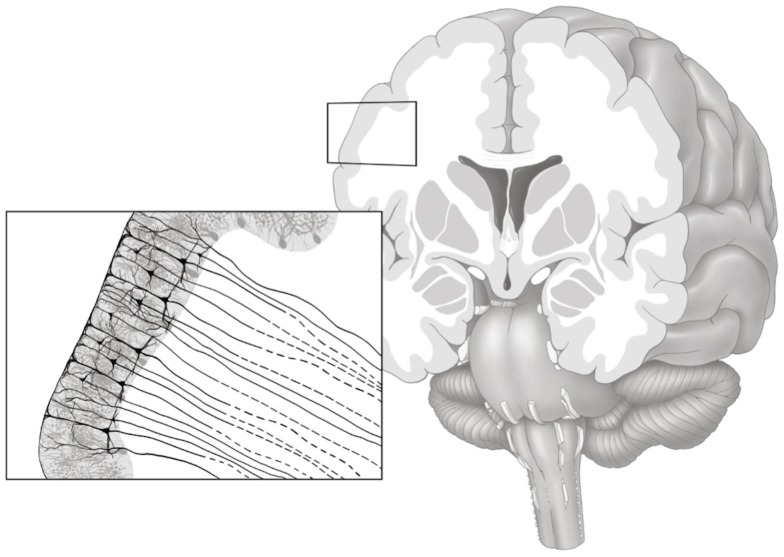
**Gray matter axonal connectivity maps quantifies a count of axonal pathways entering and exiting the gray matter tissue, as illustrated in this artistic representation**. The resulting voxel-based connectivity measure reflects the combination of all axonal pathways transitioning from the gray matter into white matter (axonal pathways leaving the gray matter are represented with dashed lines).

**Figure 2:**
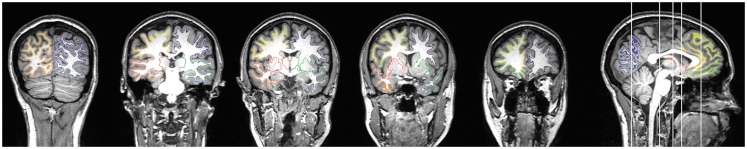
**An example of a subject’s gray and white matter transition shell is demonstrated in the color-coded voxels, where each color corresponds to the closest gray matter ROI in accordance with the Lausanne anatomical atlas**.

The purpose of the gray matter connectivity maps (henceforth denoted as GMAC) is to denote a measure of axonal pathways entering and exiting the gray matter tissue, as demonstrated in Figure 1. Thus, to quantify regional cortical connectivity, a shell was constructed to represent the voxel-layer in the transition between gray and white matter (Figure 2). For each voxel in the shell, the closest gray matter ROI was identified through a proximity-voting algorithm. In case of a draw, the assignment of the closest ROI was a random choice between the two or more equally close ROIs. The next step involved the evaluation of the connectivity of each voxel in the shell, which was accomplished by summing the number of fibers arriving at this voxel when each ROI was seeded (except for the ROI corresponding to the gray matter region immediately adjacent to that voxel, to avoid overestimating cortical connectivity). Since DTI tractography does not represent directionality of fibers, fibers traversing each voxel could represent fibers traveling either to or from the adjacent gray matter. The resulting image is a voxel-based count of regional connectivity in standard stereotaxic space, thus amenable to any form of voxel-based statistical analysis.

Below, we describe in detail each preprocessing step. We also provide a description and links to the scripts necessary to construct GMAC.

#### White matter fiber tract reconstruction

DICOM images were converted to NIfTI format (with extraction of diffusion gradient directions) using the software dcm2nii, part of the software suite MRIcron (17)^4^. The package FSL’s Diffusion Toolkit (FDT) (18)^5^ was used for preprocessing diffusion-weighted images and for diffusion-tensor estimation (13, 19). The images underwent eddy current correction through affine transformation of each DWI to the base *b* = 0, T2-weighted image.

Structural connectivity was obtained by applying FDT’s probabilistic method for fiber tracking (13, 18, 20). Probabilistic tractography was performed on diffusion data after voxel-wise calculation of the diffusion tensor. FDT’s BEDPOST was used to build default distributions of diffusion parameters at each voxel (18). Probabilistic tractography was estimated by applying FDT’s probabilistic method of fiber tracking (13), i.e., FDT’s BEDPOST was used to build default distributions of diffusion parameters at each voxel, each time evaluating the samples to generate a probabilistic distribution, which is used to build a posterior of the streamline location of the streamline location. Subsequently, tractography analyses were run using FDT’s probtrackx with default parameters, namely 5000 individual pathways drawn through the probability distributions on principle fiber direction, curvature threshold set at 0.2, 200 maximum steps, step length 0.5 mm, and distance correction (default settings). Of note, while probabilistic tractography is not the only alternative to attempting to resolve fiber crossing, with other examples being diffusion spectral imaging (21) and diffusional kurtosis imaging (22), it is likely the most appropriate method for a dataset with a limited number of diffusion directions.

Cortical seed regions for tractography were obtained from an automatic segmentation process employing FreeSurfer (15)^6^ applied to T1-weighted images. This process subdivides the human cerebral cortex into sulco-gyral-based cortical and subcortical ROIs by automatically assigning a neuroanatomical label to each location on a cortical surface model based on probabilistic information estimated from a manually labeled training set [the Lausanne anatomical atlas, distributed as part of the Connectome Mapping Toolkit (9)] (see text footnote 3), yielding 82 ROIs in the subjects’ native T1-weighted space (41 regions in each hemisphere). All processed images were visually inspected to ensure cortical segmentation quality.

The ROIs were transformed into each subject’s DTI space using an affine transformation obtained with FSL’s FLIRT (16). Probabilistic tractography was performed using the non-diffusion (B0) image as an inclusion mask. The seed masks were composed of the voxels from the gray and white matter shell corresponding to the each one of the 82 cortical ROIs in diffusion space. Each ROI shell was seeded independently (with the B0 image set as an inclusion mask, with no waypoint or termination masks). Thus, after seeding each ROI, a voxel-based map of probabilistic connections from each ROI was obtained as a three-dimensional volume and, in each volume, the number of voxel-based streamlines in each one of the other ROI shells was assessed as explained below.

#### Gray–white matter transition shell

We used the white matter mask and the gray matter ROIs generated by the cortical parcellation step to construct a shell representing the voxel-layer in the transition between gray and white matter. First, we united all gray matter ROIs into one single gray matter mask. Then, we employed a proximity-voting algorithm whereby, for each voxel in the white matter mask, a search was performed to assess which (if any) gray matter ROI was in contact with each one of the possible six sides of the voxel. If at least one side was in contact with a gray matter ROI, this white matter voxel was then included in the shell. The gray matter ROI corresponding to this voxel shell was defined as the gray matter ROI accounting for the majority of sides of the voxel (in a voting system). An example of a transition shell is demonstrated in Figure 1. The source code used for generating a shell can be observed in the Appendix below. The source code will also be available for download at http://www.mccauslandcenter.sc.edu/CRNL/ once this article has been peer reviewed and accepted for publication.

#### Gray matter axonal connectivity maps

For each voxel in the transition shell, we counted the number of tractography streamlines traversing that voxel when all other ROIs were seeded. Specifically, if the voxel being analyzed corresponded to ROI #1 (i.e., was in the transition between the gray matter from ROI#1 and white matter), we counted the number of tractography streamlines traversing that voxel when all ROIs #2 to #82 where seeded (i.e., all other ROIs). Each voxel’s resulting number of streamlines *f* was then log transformed as *f* ′ = log(*f* + 1). Finally, a within subject normalization was performed as *F* = [*f* ′ − *f* ′(min)]/[*f* ′(max) − *f* ′(min)]; where *F* is the resulting normalized voxel-based connectivity value and *f* ′(min) and *f* ′(max) are the minimal and maximal non-zero log-transformed voxel values (across the entire brain) for that subject.

Finally, the GMAC was then transformed into the subject’s native T1 space and subsequently into stereotaxic MNI space using an affine transformation obtained with FSL’s FLIRT (16).

## Results

Across all subjects, the average number of voxels in the gray–white matter transition shell was 220,018 ± 7122, which corresponded to 3 ± 0.1% of the total number of voxels in the spatially normalized T1-weighted image. The number of voxels in each ROI ranged from 9,306 ± 756 (largest ROI) to 214 ± 44 (smallest ROI).

**Figure 3:**
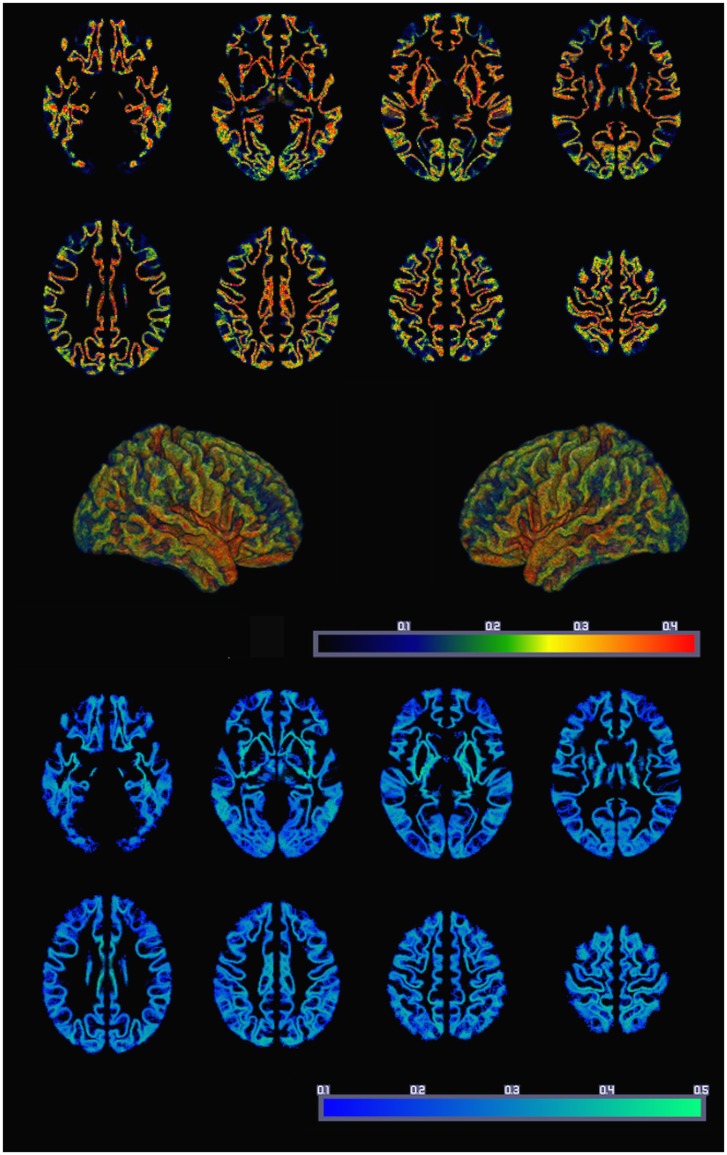
**The average whole-brain GMAC from 18 healthy adults is demonstrated in the upper panel**. Each voxel is colored in accordance with the resulting normalized voxel-based connectivity value, as demonstrated in the color bar. The middle panel illustrates a three-dimensional reconstruction of the average GMAC maps. The lower panel demonstrates the voxel-wise GMAC SD.

The resulting average whole-brain GMAC can be appreciated in Figure 3. This figure also demonstrates the voxel-based SD of GMAC. Based on visual inspection, the somatosensory cortex and the temporal and frontal opercula demonstrated a relatively higher voxel-based number of streamline counts compared with adjacent regions.

**Figure 4:**
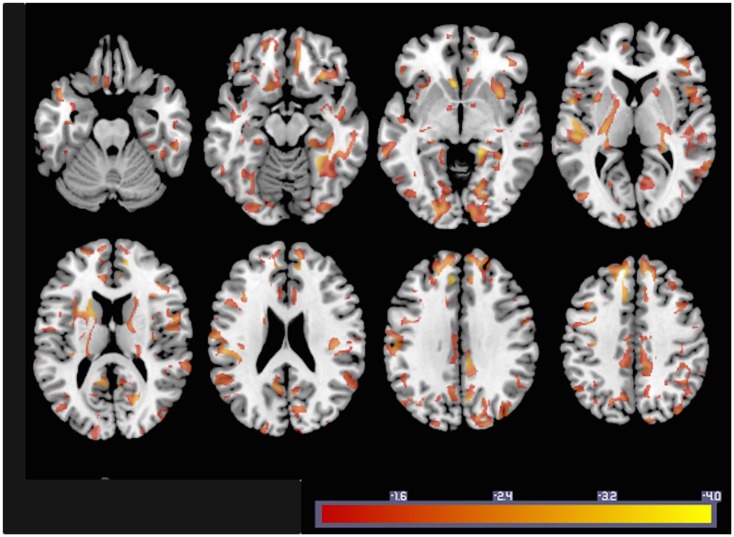
**To exemplify GMAC usability, the statistical results from a voxel-based correlation between individual GMAC (smoothed with an isometric 8 mm Gaussian kernel) and age are shown here**. Areas color-coded in “hot” represent those with a negative correlation between GMAC and age with a statistical *z* score less than −1.

In order to illustrate how GMACs can be used to investigate neurobiological phenomena, we performed a voxel-based analysis assessing the statistical relationship between gray matter connectivity and age. GMAC are immediately compatible with several well-established voxel-based tools, and we used the software NPM, part of the software package MRIcron (17)^7^ to evaluate the voxel-based correlation coefficient between age and gray matter connectivity. The results from this analysis (Figure 4) demonstrate areas with a statistical decrement in connectivity associated with older age.

## Discussion

In this study, we described a new methodology that allows for the quantitative evaluation of regional gray matter structural connections. This method has two advantages: First, GMAC provide a measure of gray matter connectivity that is largely independent from *a priori* anatomical parcellations, thus permitting a more detailed and fine-grained analysis of regional connectivity changes, without the limits imposed by the boundaries of ROIs. Second, GMAC is a voxel-based map of gray matter axonal projections in standard space, therefore amenable to statistical voxel-based analysis, which can be performed using any of the several packages for voxel-based statistical analyses that are popular in the neuroimaging community, such as, for example, NPM, SPM, and FSL.

In order to illustrate this last topic, the simple voxel-based correlation with age demonstrated a rich pattern of decrement in connectivity with older age. While the purpose of this study is to propose a new method, instead of providing an in-depth evaluation of the neurobiology of aging, the results from this correlation are in accordance with previous findings suggesting widespread reduction in white matter in healthy aging (23, 24). More importantly, these results provide an example of the utility of the GMAC, which can help reveal a finer grained pattern of connectivity decrement, which could have been missed by ROI analyses when the values of all included voxels are averaged and regional changes, within ROI effects, are possibly overlooked.

Another practical utility of GMAC is the anatomical display of the connectivity patterns through the use of volume or surface rendering software. Since GMAC are voxel-based images, they are compatible with several three-dimensional volume reconstruction programs, such as, for example, MRIcro (25), FSLView (26), MRIcroGL^8^, BrainNetViewer (27), and MRIcroS^9^. This feature will enable the visualization of regional connectivity patterns that are difficult to discern from two-dimensional connectome data.

We believe that an important application of GMAC will be its evaluation in the context of brain damage, akin to voxel-based lesion-symptom mapping (VLSM). Our group recently demonstrated that neuronal loss may affect remote areas after tissue necrosis from stroke (28), leading to gray matter disconnection, even though this pattern is largely invisible to many quantitative imaging modalities. In fact, disconnection syndromes are a prominent clinical phenomenon in neurology, but the quantification of structural disconnection has been hitherto elusive due to limitations in direct connectivity measures. It is only through the use of comprehensive connectome mapping that it is now possible to appreciate the extent of remote axonal loss and its clinical relevance. At the moment, there are no methods that provide a voxel-based whole-brain map of gray matter connectivity, and GMAC will fill this gap. Moreover, another practical utility of these methods are the use of gray–white matter transition shells to better define the white matter boundaries from ROIs, for example, from functional MRI studies, thus permitting a better evaluation of regional axonal connectivity related to functional areas.

Compared with regular connectome mapping, the main disadvantages of GMAC are: first, the absence of information regarding pairwise connections, i.e., if GMAC are constructed from the entire connectome, GMAC provide a measure of regional gray matter connectivity but it does not provide information of the target or the origin of the fibers reaching that voxel. A simple strategy to overcome this problem would be to calculate the GMAC based on fibers obtained from seeding only a limited number of ROIs; for example, how much voxel-based connectivity is there in the hippocampus when only the anterior cingulate is seeded. This later approach is akin to the previously described connectivity parcellation maps, as elegantly demonstrated for connectivity-based segmentation of the thalamus (29, 30) or Broca’s area (31).

The second limitation is the inability to calculate graph-based measures from the GMAC, since link-based information is not included. For network architecture measures, the use of connectome matrices is suggested and preferred.

Considering that GMACs are constructed based on data from DTI tractography, cortical connectivity may also be related to how accurate fiber tracking occurs in the adjacent white matter, with a higher connectivity observed in regions that are immediately adjacent to, or overlying, large white matter pathways; while lower connectivity occurs in areas overlying white matter regions where tracking is less accurate, such as locations with extensive fiber crossings. Furthermore, the approach presented here is a relatively conservative, not taking into account regional microanatomy. A less conservative approach would entail assessing a weighted average of the voxels in relationship with its neighbors, but this approach could lead to artificially high numbers since voxel boundaries may span over anatomical boundaries.

In summary, in this study, we introduce a practical and readily accessible approach to generate whole-brain maps of gray matter connectivity. GMAC maps provide information that is not exclusively limited to *a priori* anatomical parcellations and expand connectomics research to a voxel-based metric that can be analyzed using conventional voxel-based statistical packages. As such, any form of voxel-based statistical tests can be applied to GMACs, including analyses evaluating continuous data, as well as thresholded approaches that are analogous to lesion-symptom mapping. There are no other currently available methods that quantify the same biological features that are measured by GMAC. For this reason, the applicability of GMAC is potentially vast, encompassing the study of neurobiological phenomena that are directly or indirectly supported by the integrity of regional gray matter connectivity.

## Conflict of Interest Statement

The authors declare that the research was conducted in the absence of any commercial or financial relationships that could be construed as a potential conflict of interest.

## Footnotes

^1^http://www.fz-juelich.de/inm/inm-1/EN/Forschung/_docs/SPMAnatomyToolbox/SPMAnatomyToolbox_node.html

^2^http://surfer.nmr.mgh.harvard.edu/

^3^http://www.connectome.ch

^4^http://www.mccauslandcenter.sc.edu/mricro/mricron/dcm2nii.html

^5^www.fmrib.ox.ac.uk/fsl

^6^http://surfer.nmr.mgh.harvard.edu/

^7^http://www.mccauslandcenter.sc.edu/mricro/mricron/

^8^http://www.mccauslandcenter.sc.edu/mricrogl/

^9^http://www.nitrc.org/projects/mricros/

## Appendix

### Matlab source code to generate a gray–white matter transition shell

function roi_shell = create_roi_shell(roi_data, wm_data)

%% “create_roi_shell” propagates ROI labels to 1-voxel thick shell

%

% create_roi_shell(roi_data, wm_data)

%

% For a given ROI data volume, generate an “roi-shell,” where every voxel

% greater than zero in the white matter mask data volume that also

% neighbors a region is set equal to the label of the ROI region of which

% it shares the most adjacent voxels. If a given shell voxel has two or

% more maximal ROI neighbors, the voxel defaults to the ROI with the

% lowest value.

%

% INPUT

% roi_data – an ROI data volume with regions labeled as succesive

%  integers

% wm_data – a mask indicating white matter where any value greater

%  than zero will be treated as white matter

%

%%%%%%%%%%%%%%%%%%%%%%%%%%%%%%%%%%%%

% remove any of the white matter masks that happens to overlap with the

% ROIs, we want the very outer bound of the ROIs

wm_data(roi_data > 0) = 0;

% convolve roi_data where they intersect with wm_data, anything greater

% than zero in the result is a white matter voxel that neighbors an ROI

kernel = [[[0 0 0];[0 1 0];[0 0 0]];

   [[0 1 0];[1 0 1];[0 1 0]];

   [[0 0 0];[0 1 0];[0 0 0]]];

kernel = reshape(kernel,3,3,3);

shell_mask = (convn(roi_data,kernel,‘same’) > 0) & (wm_data > 0);

% create 6 neighbor kernels designed to extract the value of a specific

% neighbor voxel using convolution

n_kernel = zeros(6,3,3,3);

n_kernel(1,2,2,1) = 1;

n_kernel(2,1,2,2) = 1;

n_kernel(3,2,1,2) = 1;

n_kernel(4,2,3,2) = 1;

n_kernel(5,3,2,2) = 1;

n_kernel(6,2,2,3) = 1;

% create a representation of the shell image where the 4th dimension is

% designed to hold possible values for each of the voxel’s 6 neighbors

shell_neighbors = zeros(dims(1),dims(2),dims(3),6);

for n = 1:6

 shell_neighbors(:,:,:,n) = convn(roi_data, n_kernel(n,:,:,:),‘same’);

 shell_neighbors(:,:,:,n) = shell_neighbors(:,:,:,n) .* shell_mask;

end

% we now want the mode across the last dimension of our shell_neighbors

% matrix… but we really only care about a small amount of voxels, so it

% is actually quicker to loop through the voxels that we care about

% rather than perform the operation on the entire matrix

roi_shell = zeros(size(shell_mask));

[X,Y,Z] = ind2sub(size(shell_mask), find(shell_mask));

for idx = 1:length(X)

 x=X(idx);y=Y(idx);z=Z(idx);

 roi_shell(x,y,z) = mode(nonzeros(shell_neighbors(x,y,z,:)));

end

roi_shell(isnan(roi_shell)) = 0;
